# Treatment rationale and design of the RAMNITA study: A phase II study of the efficacy of docetaxel + ramucirumab for non–small cell lung cancer with brain metastasis

**DOI:** 10.1097/MD.0000000000011084

**Published:** 2018-06-18

**Authors:** Keiko Tanimura, Junji Uchino, Nobuyo Tamiya, Yoshiko Kaneko, Tadaaki Yamada, Kenichi Yoshimura, Koichi Takayama

**Affiliations:** a Department of Pulmonary Medicine, Kyoto Prefectural University of Medicine, Kyoto; b Department of Biostatistics, Innovative Clinical Research Center, Kanazawa University, Kanazawa, Ishikawa, Japan.

**Keywords:** brain metastasis, docetaxel, non–small cell lung cancer, ramucirumab, study protocol

## Abstract

**Background:**

We described the treatment rationale and procedure for a phase II study of docetaxel plus ramucirumab for non–small cell lung cancer (NSCLC) patients with brain metastasis (RAMNITA study: University Information Network Clinical Trials Registry identification no. [UMIN]: 000024551). Combination therapy of angiogenetic inhibitor with chemotherapy improved the outcome of patients with brain metastasis in previous reports; however, the efficacy of ramucirumab, a vascular endothelial growth factor receptor-2 monoclonal antibody, for brain metastasis has not been shown.

**Patients and methods:**

This RAMNITA study is a prospective, multicenter, open-label, single-arm phase II study designed to evaluate efficacy and safety of docetaxel and ramucirumab for advanced NSCLC patients with brain metastasis. Eligible patients will receive docetaxel (60 mg/m^2^) and ramucirumab (10 mg/kg) every 21 days until disease progression. The primary endpoint is progression-free survival (PFS), and secondary endpoints are overall survival, intracranial PFS, response rate, and safety. Sixty-five participants will be recruited from September 2017 to December 2019 and followed up for 1 year after final registration. The results from this study may suggest a treatment option for brain metastasis in NSCLC.

**Ethics:**

The protocol was approved by the institutional review board of each study center. Written informed consent will be obtained from all patients before registration, in accordance with the Declaration of Helsinki.

## 1 Introduction

Non–small cell lung cancer (NSCLC) is usually diagnosed at an advanced stage of the disease, and brain metastasis is a common complication in NSCLC patients, with >10% of patients with NSCLC presenting with brain metastasis at their first hospital visit ^[1,2]^ and 30% to 40% of patients with NSCLC developing brain metastasis during the course of the disease.^[3]^ Although efficacy of chemotherapy for brain metastasis is limited, radiological therapies, including stereotactic radiosurgery (SRS) and whole brain radiotherapy, or surgical resection may be used for local control of brain metastasis.

In cancer, tumor angiogenesis owing to overexpression of angiogenetic factors, such as vascular endothelial growth factor (VEGF) receptor, create an abnormal tumor microenvironment characterized by hypoxia and acidosis, and interstitial hypertension owing to vascular hyperpermeability, which reduces drug penetration into tumors. Antiangiogenetic agents can decrease tumor vascular permeability and interstitial fluid pressure by inhibiting of tumor angiogenesis, and thereby improve the efficacy of coadministered anticancer drug(s).^[4]^

Previous research revealed angiogenesis via the VEGF pathway is involved in the formation of brain metastasis. Subset analysis of AVAiL trial data showed that bevacizumab combined with platinum-doublet chemotherapy significantly decreased brain metastasis development.^[5]^ Furthermore, bevacizumab combined with cytotoxic agents improved the survival of patients with newly detected brain lesions.^[6,7]^

Ramucirumab is a human recombinant IgG1 monoclonal antibody that specifically binds to the extracellular domain of VEGF receptor-2 with high affinity, preventing the binding of VEGF ligands and receptor activation.^[4]^ The REVEL study was a global, randomized, placebo-controlled, double-blind, multicenter phase III study comparing docetaxel plus ramucirumab combination treatment with docetaxel treatment (docetaxel plus placebo) in patients with stage IV NSCLC who showed disease progression after platinum-based therapy. This study showed that second-line docetaxel plus ramucirumab combination treatment of patients with stage IV NSCLC improves progression-free survival (PFS), overall survival (OS), and response rate; however, the efficacy of ramucirumab for brain metastasis remained unclear.^[8,9]^ The current trial is designed to evaluate the efficacy and toxicity of a docetaxel plus ramucirumab regimen as a treatment for NSCLC with brain metastasis.

## 2 Patients and methods

### 2.1 Study design

**Figure 1:**
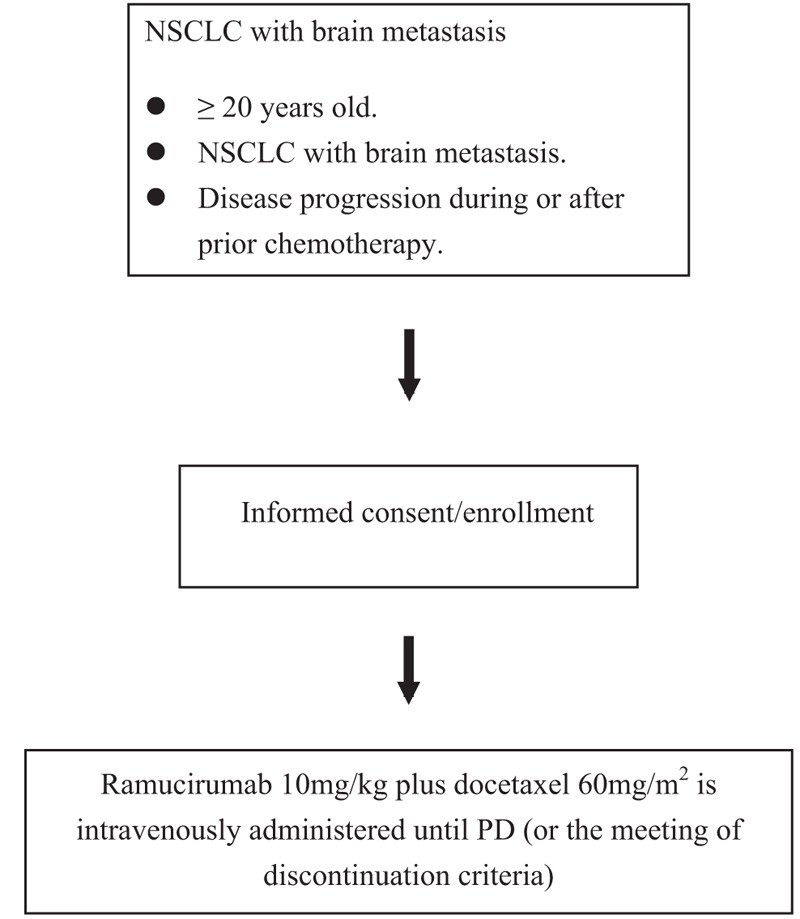
Study flow chart. NSCLC = non–small-cell lung cancer.

The RAMNITA study is an open-label, single-arm trial of NSCLC with brain metastasis. Figure 1 depicts a flow chart of the study. The aim of this study is to investigate the efficacy and safety of ramucirumab with docetaxel in patients with advanced or recurrent NSCLC who have brain metastasis. Patients are registered in this study after independent review by the Data Center of the Clinical Research Support Centre Kyushu, where the potential subjects are screened against the inclusion and exclusion criteria. At least annual independent monitoring is planned, in accordance with the Japanese clinical trial guideline. We plan to recruit 65 patients from September 2017 to December 2019. The observational period is 1 year from time of final registration. The primary endpoint is PFS, and secondary endpoints are OS, intracranial PFS, response rate, and safety.

### 2.2 Treatment

Intravenous administration of ramucirumab 10 mg/kg plus docetaxel 60 mg/m^2^ on day 1 of a 3-week cycle will be continued until disease progression or fulfillment of the criteria of treatment cessation. No dosage adjustment according to age, body weight, sex, ethnicity, and smoking status is warranted.

This study has been conducted in compliance with the principles of the Declaration of Helsinki and registered in the University Hospital Medical Information Network database (UMIN000024551).

### 2.3 Key eligibility criteria

Key eligible criteria are listed below.

<Inclusion criteria>

(1) Histological or cytological confirmation as advanced NSCLC
(2) Asymptomatic brain metastasis is present. SRS (including Gamma Knife or Cyber Knife) before enrollment is allowed.
(3) A history of chemotherapy
(4) Performance status (ECOG) is 0 or 1.
(5) Age ≥20 years
(6) Bone marrow, coagulation factor activity, and organ functions have all been confirmed as normal.
(7) Life expectancy of at least 3 months
(8) If sexually active, patients must be postmenopausal, surgically sterile, or using effective contraception.
(9) Other cancers must have been treated curatively and followed without evidence of recurrence for at least 3 years before acquisition of consent.
(10) Provision of written informed consent

<Exclusion criteria>

(1) Receiving other anticancer therapy currently
(2) Presenting symptoms caused by brain metastasis
(3) History of whole brain radiotherapy or surgical resection for brain metastasis
(4) Presence of meningeal metastasis
(5) Bleeding into the brain metastasis or other central nervous system hemorrhage within 21 days before acquisition of consent
(6) Intratumor cavitation or major blood vessel invasion or encasement by cancer
(7) Arterial thromboembolic events, venous thromboembolism events, or serious bleeding complications within 6 months before acquisition of consent
(8) Other serious complications
(9) Patients who are pregnant, nursing, or possibly pregnant
(10) Uncontrolled metabolic disorder, including diabetes mellitus
(11) Active or uncontrolled clinically serious infection

### 2.4 Patient registration

After the eligibility criteria have been met and the patients have provided informed consent, eligible patients will be registered and the planned treatment will be initiated by the investigators. Accrual began in May 2017 and should continue for 3 years.

### 2.5 Evaluation of response and safety

Patients will be administered docetaxel and ramucirumab every 3 weeks beginning on day 1 and continue until disease progression or the fulfillment of the discontinuation criteria. Tumor assessments by imaging (brain magnetic resonance imaging [MRI] tumor and thoracoabdominal computed tomography) should be performed as scheduled every 6 weeks (±2 weeks) during 8 months following protocol treatment commencement, and every 9 weeks (±2 weeks) thereafter, until progressive disease is documented on imaging.

The antitumor effect is evaluated according to the “New response evaluation criteria in solid tumors: Revised RECIST guideline (version 1.1)—Japanese JCOG edition.”

For assessment of brain metastasis, “Brain Metastases from Solid Tumors: Implementing Response Assessments” (https://www.parexel.com/files/5214/0422/3830/MI_Brain_Metastases_White_Paper_JUN_14.pdf) will be used. Target lesions and nontarget lesions defined on gadolinium-enhanced MRI with a 3-mm maximum slice thickness.

### 2.6 Statistical design

From an analysis of JVCG phase II study data from patients treated by ramucirumab-docetaxel, median PFS and OS were reported to be 5.22 months (95% CI: 3.52–6.97 months) and 15.15 months (95% CI: 12.45–26.55 months), respectively.^[10]^ On the contrary, life expectancy for patients with brain metastasis was poor, with a median survival of only 3.4 months.^[11]^

As mentioned above, the threshold median PFS and expected median PFS are estimated to be 3.0 and 5.3 months, respectively. Under these conditions and assuming a 2-sided significance level of 5% and power of 75% from 1 sample nonparametric tests for a median survival time, 61 subjects are required (Nagashima K. A sample size estimation tool for one sample nonparametric tests for a median survival time [Internet]. March 21, 2016 [cited 2016 September 25]; Available from: http://nshi.jp/ejs/onesurvmst/). Allowing for dropouts, 65 subjects will be enrolled.

### 2.7 Ethics

The trial received ethical approval from the Ethics Committee of Kyoto Prefectural University of Medicine, Kyoto, Japan (number: ERB-C-694-3, the last edition ver 2 04/Aug/2017). The trial is subject to the supervision and management of the Ethics Committee.

## 3 Discussion

Although the REVEL study showed efficacy and tolerability of the treatment regimen with starting dose of ramucirumab 10 mg/kg and docetaxel 75 mg/m^2^, the permissible starting dose of docetaxel for East Asian patients is 60 mg/m^2^.^[9]^ Therefore, we are using this dose in our trial.

For assessment of the tumor response, we are using 2 sets of guidelines: Response Evaluation Criteria in Solid Tumors (RECIST) for extracranial lesions, and “Brain Metastases from Solid Tumors: Implementing Response Assessments” for intracranial lesions. Patients who received only SRS or do not need treatment for local control of their brain tumors are included in this study; therefore, the brain lesions of most of the participants are <3 cm in diameter and amenable to SRS. Brain tumors initially >1 cm in diameter can be evaluated according to the RECIST guideline. On the contrary, “Brain Metastases from Solid Tumors: Implementing Response Assessments” is suitable for evaluating brain tumors >5 mm in diameter.

## 4 Conclusion

To the best of our knowledge, this study is the first clinical trial of ramucirumab combination therapy for NSCLC with brain metastasis. Our study may provide new evidence about brain metastasis treatment in patients with NSCLC.

## Acknowledgments

The authors thank the patients, their families, and all investigators involved in this recent study.

## Author contributions

**Conceptualization:** Keiko Tanimura, Junji Uchino.

**Formal analysis:** Kenichi Yoshimura.

**Funding acquisition:** Junji Uchino.

**Investigation:** Keiko Tanimura, Nobuyo Tamiya, Yoshiko Kaneko, Tadaaki Yamada.

**Supervision:** Koichi Takayama.
